# Editorial: The Hybrid Operating Room in Modern Thoracic Surgery

**DOI:** 10.3389/fsurg.2021.725897

**Published:** 2021-07-23

**Authors:** Calvin S. H. Ng, William S. Krimsky, Kazuhiro Yasufuku

**Affiliations:** ^1^Department of Surgery, The Chinese University of Hong Kong, Hong Kong, China; ^2^Gala Therapeutics, Menlo Park, CA, United States; ^3^Division of Thoracic Surgery, Toronto General Hospital, University of Toronto, University Health Network (UHN), Toronto, ON, Canada

**Keywords:** hybrid operating room, navigation bronchoscopy, video-assisted thoracic surgery, pulmonary nodule, ablation, microwave, cone-beam CT, 3D-mapping

Chest clinicians are increasingly being called to manage small indeterminate pulmonary nodules. This global trend is likely the result of a combination of different factors, including the increase in availability of high-end imaging, the increase in awareness of the need for early diagnosis of lung pathologies, particularly lung cancer, and perhaps most importantly, due to adoption of lung cancer screening by computed tomography (CT) scanning in more and more countries.

One major challenge of managing small, indeterminate pulmonary nodules is the need for a reliable method or technique to accurately localize and reach the target lesion or lesions so as to be able to:

- to perform a diagnostic biopsy
- to mark or label the target for surgical resection
- And in select cases, to non-surgically treat it.

Compared with many other organs, such as esophagus and colon, the development of solutions to manage these challenges in the lungs have been slow.

The development of hybrid operating suite is a small, albeit critical step in the management of small indeterminate pulmonary nodules. The ability to have real-time high definition imaging during the endoscopic or surgical procedure has revolutionized and improved current techniques of lung navigation and nodule localization. In this edition, we cover the use of electromagnetic navigation bronchoscopy (ENB) technology and percutaneous lung nodule localization in the hybrid operating room, as well as discuss the value and advantages of these integrated approaches. Furthermore, several authors present their future perspective on therapeutic roles of the hybrid theater in guiding surgery and local therapies.

Zhao et al. described electromagnetic navigation bronchoscopy utilization in hybrid theater and summarized in a mini-review its uses in biopsy, nodule localization, and transbronchial local therapy. Although the use of cone-beam CT scan in conjunction with ENB for lung nodule biopsy is not entirely novel as shown by the NAVIGATE trial, due to limited availability and costs, cone-beam CT is far from being routinely used. The integration of ENB into a hybrid operating suite also requires special considerations during installation and usage to avoid interference that could lead to inaccuracies in navigation. Another important application of ENB in hybrid theater would be dye or contrast labeling for assisting pulmonary resection. Marking through the endobronchial route could potentially reduce dye dispersion and have lower incidence of pneumothorax when compared with other percutaneous marking techniques. Clearly, the role of cone-beam CT to guide precise ENB navigation and to confirm successful marking, particularly in provide information on the relative position of the dye in relation to the nodules cannot be underestimated. Zhao et al.'s article ends with what may become of combining navigation bronchoscopy and hybrid theater in delivering alternative therapies to ablate pulmonary nodules.

Leow and Chao from Taiwan presented their formidable patient-tailored experience of image guided video-assisted thoracoscopic surgery (iVATS) within hybrid operating room environment through different marking approaches (single vs. double-marker) and access routes (percutaneous technique with cone-beam CT imaging vs. ENB). Their success rate of 95.9% for localizing 174 consecutive nodules with mean nodule size of 8.3 mm and mean distance from pleural surface of 9.4 mm was admirable. Perhaps the most interesting aspect of their study and points for learning are the very few failed localization cases. Of note, technical complications such as pneumothorax and microcoil dislodgement represented 57% of their failures, while the robotic C-arm cone-beam CT machine failure accounted for the rest. This is a poignant reminder that as we become more technologically dependent in our surgical approaches as a way to better tailor therapy for our patients and improve outcomes, at the same time we are to a certain extent at the mercy of the machines that have become our operative partners.

Kothapalli et al. first reviewed the current techniques for localization of pulmonary nodules using CT, and then weighed up the benefits and limitations of the hybrid operating room approach. One important benefit of the one stop-shop approach of performing the localization in the hybrid room is the reduced delay between localization and surgery. Shortening this period minimizes potential life-threatening complications of the localization procedure from developing, as well as decrease risk of marker migration or dislodgement. The trade-off would be the radiation exposure to patient and operator(s), increase costs of prolonged occupancy of an operating room and change in set up to allow the CT scan to proceed. In the latter part of their review, the value of using hybrid theater with cone-beam CT as well as future directions for research and development are discussed. One of the more exciting areas where real-time imaging can prove to be useful is for guiding resection. Once the challenge of 3D-mapping of a collapse lung is overcome, 3D-image fusion and overlay during lung resection may become a distinct possibility. They also make a very practical point about the need for education and training within thoracic surgical programs to facilitate hybrid theater procedure development.

The articles within the topic of Hybrid Operating Room in Modern Thoracic Surgery has provided the reader with great insight into current uses of intra-operative imaging in management of pulmonary nodules. In addition, we believe that it has also highlighted potential areas needing refinement in workflow, technology, and integration. As demand and interest continues to grow in this field, we look forward to exciting and rapid developments in hybrid operating room use in thoracic surgery.

## Author Contributions

All authors listed have made a substantial, direct and intellectual contribution to the work, and approved it for publication.

## Conflict of Interest

WS has intellectual property rights with Medtronic and is an employee of Gala. The remaining authors declare that the research was conducted in the absence of any commercial or financial relationships that could be construed as a potential conflict of interest.

## Publisher's Note

All claims expressed in this article are solely those of the authors and do not necessarily represent those of their affiliated organizations, or those of the publisher, the editors and the reviewers. Any product that may be evaluated in this article, or claim that may be made by its manufacturer, is not guaranteed or endorsed by the publisher.

